# Structural analysis of APOB variants, p.(Arg3527Gln), p.(Arg1164Thr) and p.(Gln4494del), causing Familial Hypercholesterolaemia provides novel insights into variant pathogenicity

**DOI:** 10.1038/srep18184

**Published:** 2015-12-08

**Authors:** J. A. Fernández-Higuero, A. Etxebarria, A. Benito-Vicente, A. C. Alves, J. L. R. Arrondo, H. Ostolaza, M. Bourbon, C. Martin

**Affiliations:** 1 Unidad de Biofísica (CSIC, UPV/EHU) and Departamento de Bioquímica y Biología Molecular, Universidad del País Vasco, Apdo. 644, 48080 Bilbao, Spain; 2 Unidade de I&D, Grupo de Investigação Cardiovascular, Departamento de Promoção da Saúde e Prevenção de Doenças Não Transmissíveis, Instituto Nacional de Saúde Doutor Ricardo Jorge, Lisboa, Portugal; 3 BioISI – Biosystems & Integrative Sciences Institute, Faculdade de Ciências, Universidade de Lisboa, Lisboa, Portugal

## Abstract

Familial hypercholesterolaemia (FH) is an inherited autosomal dominant disorder resulting from defects in the low-density lipoprotein receptor (*LDLR*), in the apolipoprotein B (*APOB*) or in the proprotein convertase subtilisin/kexin type 9 (*PCSK9*) genes. In the majority of the cases FH is caused by mutations occurring within *LDLR*, while only few mutations in *APOB* and *PCSK9* have been proved to cause disease. p.(Arg3527Gln) was the first mutation in *APOB* being identified and characterized. Recently two novel pathogenic *APOB* variants have been described: p.(Arg1164Thr) and p.(Gln4494del) showing impaired LDLR binding capacity, and diminished LDL uptake. The objective of this work was to analyse the structure of p.(Arg1164Thr) and p.(Gln4494del) variants to gain insight into their pathogenicity. Secondary structure of the human ApoB100 has been investigated by infrared spectroscopy (IR) and LDL particle size both by dynamic light scattering (DLS) and electron microscopy. The results show differences in secondary structure and/or in particle size of p.(Arg1164Thr) and p.(Gln4494del) variants compared with wild type. We conclude that these changes underlie the defective binding and uptake of p.(Arg1164Thr) and p.(Gln4494del) variants. Our study reveals that structural studies on pathogenic variants of *APOB* may provide very useful information to understand their role in FH disease.

Lipoproteins play important physiologic roles in cellular function and regulation of metabolic pathways. Apolipoprotein B-100 (ApoB100) are scaffolds of low-density lipoprotein (LDL) particles that are the major natural transporter of cholesterol and phospholipids, acting as a constant supply of cholesterol for peripheral tissues and cells^1^. ApoB100 contains multiple lipid-associating regions^2^ to adopt the required structure for binding to the LDL receptor (LDLR). Familial hypercholesterolaemia (FH) is a common genetic disorder characterized by elevated circulating LDL cholesterol, tendon xanthomas, and premature coronary heart disease^3^,^4^,^5^,^6^. Mutations in *LDLR* are the most common cause of FH, while mutations in *APOB* and *PCSK9* are rare common causes of FH^7^. So far, only a few mutations have been reported and characterized as functionally defective in *APOB*^3^,^8^,^9^,^10^,^11^,^12^. The most common mutation found in *APOB* is a single amino acid substitution of arginine for glutamine at position 3527 (p.(Arg3527Gln)), which markedly reduces the affinity for the LDLR^10^,^11^. It has been described that the highly conserved receptor binding-site is stabilized by the interaction of Arg3527 with Trp4396, and as mentioned above, replacement of Arg3527 by a Gln impairs receptor recognition^3^,^10^. Other pathogenic alterations have been described, p.(Arg3507Trp), p.(Arg3558Cys), p.(Trp4396Tyr), and recently our group described and characterize two novel mutations, p.(Arg1164Thr) and p.(Gln4494del), located in exon 22 and 29, respectively^8^. Strikingly none of these alterations reside in the consensus region of the ApoB100/LDLR binding or in the region postulated by Krisko and Etchebest^13^. Hence, structural characterization of *APOB* pathogenic variants would improve our understanding of both ApoB100 structure-function relationship and mechanisms of interaction with LDLR. The extraordinary size of ApoB100, which is a monomeric protein formed by 4,536 amino acids^14^, is thus a challenge for structural studies. LDL surface is surrounded by a single copy of ApoB100, with some regions rich in β-type structures embedded in the lipid domain of the particle^15^,^16^, while residues involved in LDLR binding are exposed to the medium^16^. It has been suggested that ApoB100 is composed of globular domains connected by flexible chains that stabilize the structure of the protein-lipid complex^17^,^18^. Analysis of the sequence suggests that ApoB100 contains five distinct alternating α-helical and β-sheet domains: NH- βα1-β1-α2-β2-α3-COOH with different lipid binding affinities^19^,^20^. Currently, no high-resolution image of any ApoB100 domains is available and different attempts have been made to elucidate LDL:ApoB structure. Among them are included, small angle neutron scattering of lipid-free ApoB100 which describes the modular nature of the protein, with ordered domains connected by flexible linkers^21^; small-angle X-ray scattering which models the LDL core at low-resolution^22^, and cryomicroscopy for single particle reconstruction^23^. Infrared (IR) spectroscopy complements the information obtained by other methodologies, providing information related to size and density of LDL and, also about the secondary structure content of ApoB100^24^,^25^. Therefore, IR spectroscopy is suitable to have a rapid estimation whether an *APOB* variant produce significant changes in the overall structure of lipoprotein particle.

In the current study we have analysed the particle size of LDL carrying wt ApoB100, and from heterozygote patients carrying p.(Arg3527Gln) (c.10580G>A), p.(Arg1164Thr) (c.3491G**>**C) and p.(Gln4494del) (c.13480_13482delCAG) variants by dynamic light scattering (DLS) and electron microscopy (EM) and their secondary structure by IR spectroscopy. We have found differences both in particle size and in the ApoB100 secondary structure between the mutant particles compared with wt ApoB100 particles that may underlie the defective binding and uptake of LDL carrying these ApoB100 variants leading to FH.

## Results

### Impaired binding capacity of p.(Arg1164Thr) and p.(Gln4494del) ApoB100 variants

**Figure 1:**
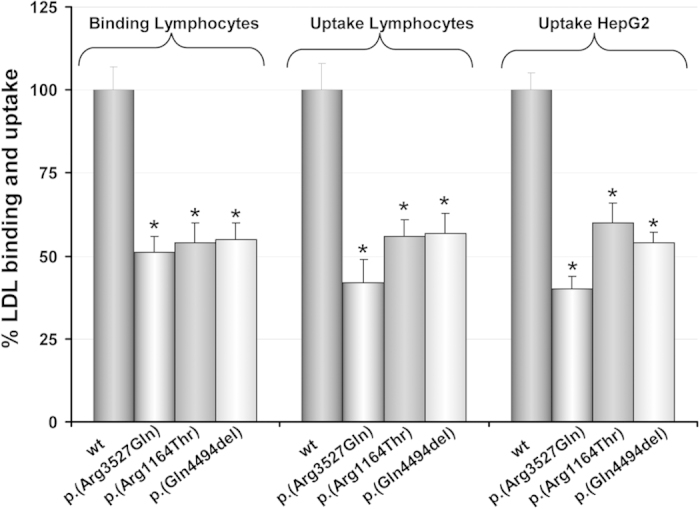
Binding of FITC-LDL in lymphocytes and uptake of FITC-LDL in lymphocytes and HepG2. Binding of FITC-LDL was assessed by incubation of lymphocytes with 20 μg/mL labelled-LDL for 4 h at 4 °C. For analysis of FITC-LDL uptake, lymphocytes or HepG2 cells were incubated for 4 h at 37 °C with 20 μg/mL FITC-LDL. 10,000 cells were acquired in a FACScalibur. The values represent the mean of triplicate determinations (n = 3); error bars represent ± SD. *p < 0.025 compared with wt ApoB100 (Student’s t-test, two tailed).

As described before by Alves and colleagues^8^, binding of p.(Arg3527Gln), p.(Arg1164Thr) and p.(Gln4494del) in lymphocytes resulted diminished when compared with wt ApoB100 (Fig. 1). LDL uptake showed impaired LDL uptake by the pathogenic variants of about 50% compared with wt (Fig. 1). Deficient LDL uptake of the LDL containing ApoB100 variants by HepG2 cells was also confirmed, being LDL uptake similar to that determined in human lymphocytes (Fig. 1).

**Figure 2:**
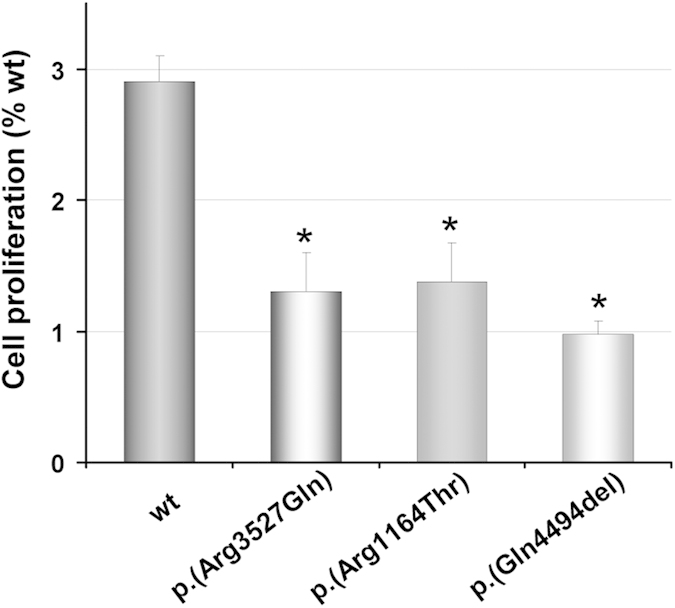
U937 cell proliferation assay with wt or LDL containing ApoB-100 variants. Cells were incubated for 48 h with LDL and proliferation was assessed by CellTiter 96wAQueous Non-Radioactive Cell Proliferation Assay. The values represent the mean of triplicate determinations (n = 3); error bars represent ± SD. *p < 0.025 compared with wt ApoB100 (Student’s t-test, two tailed).

Next, we confirmed the impaired binding capacity of p.(Arg3527Gln), p.(Arg1164Thr) and p.(Gln4494del) variants with the U937 cells proliferation assay which is a reference method to determine functional assays in APOB^26^. Cells growing in the presence of LDL carrying p.(Arg3527Gln), p.(Arg1164Thr) or p.(Gln4494del) variants showed ~1/3 growth compared with wt LDL (Fig. 2).

### LDL size analysis

**Figure 3:**
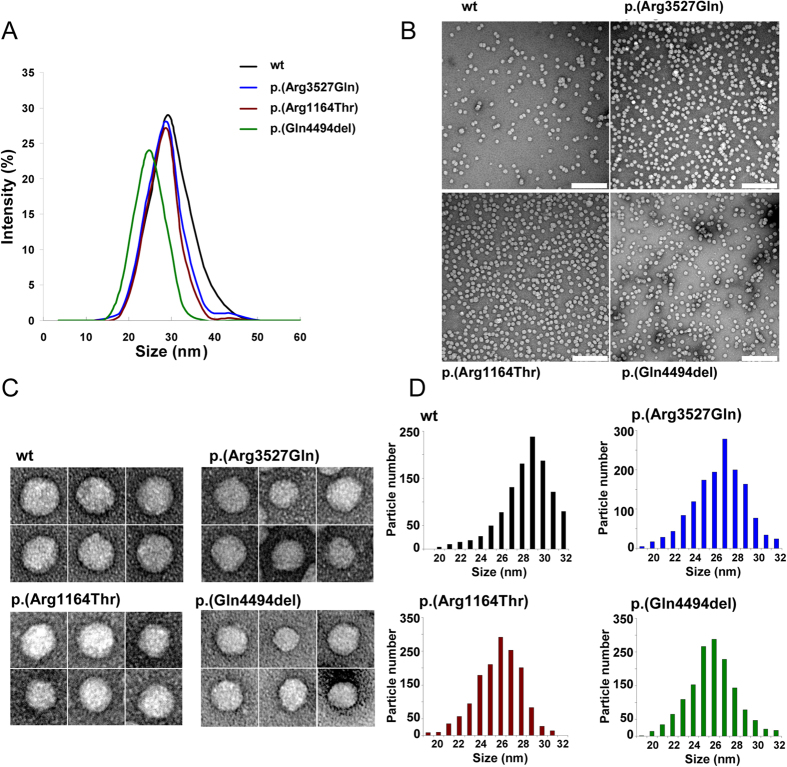
Different LDL size distribution among ApoB-100 variants. (**A**) Size distribution of isolated LDL carrying wt, p.(Arg3527Gln), p.(Arg1164Thr) and p.(Gln4494del) variants determined by DLS; (**B**) electron-micrographs of wt, p.(Arg3527Gln), p.(Arg1164Thr) and p.(Gln4494del) variants at low resolution showing a homogeneus particle population; (**C**) selected individual LDL particles at higher magnification showing and (**D**) Frequency histograms showing particle size distribution of wt, p.(Arg3527Gln), p.(Arg1164Thr) and p.(Gln4494del) variants. Particle size was determined as described in Materials and Methods. LDL size distribution in D was measured as Feret diameter calculated from 1600 particles.

Peak diameters of LDL particle carrying wt ApoB100, p.(Arg3527Gln), p.(Arg1164Thr) and p.(Gln4494del) were determined by DLS (Fig. 3A). The mean LDL diameter of the wt ApoB100-LDL was 29.1 ± 0.2 nm and, the diameters of p.(Arg3527Gln), p.(Arg1164Thr) and p.(Gln4494del) -LDL were 27.8 ± 0.3 nm, 27.9 ± 0.6 nm and 25.2 ± 0.9 nm, respectively.

LDL carrying wt ApoB100 or p.(Arg3527Gln), p.(Arg1164Thr) or p.(Gln4494del) variants were examined by NS-EM for morphology and size estimation. All images examined were approximately circular, consistent with a spherical shape (Fig. 3B,C). For wt LDL, 75% of the selected 1600 particles were in the diameter range of 27–31 nm, with the peak population (21%) at 29 nm (Fig. 3D). However, electron micrographs of patients LDL with apoB variants p.(Arg3527Gln), p.(Arg1164Thr) and p.(Gln4494del) showed smaller diameters. Accordingly, for p.(Arg3527Gln) and p.(Arg1164Thr) variants most of the particles (~78%) were in the diameter range of 25–29 nm, with the peak population (~ 20%) at 27 nm (Fig. 3D). However, electron micrographs of p.(Gln4494del)-LDL showed that most (81%) of the selected particles were between 24–27 nm in diameter with the peak population (77%) at 25.5 nm (Fig. 3D).

### Differences in secondary structure content among ApoB100 variants

**Figure 4:**
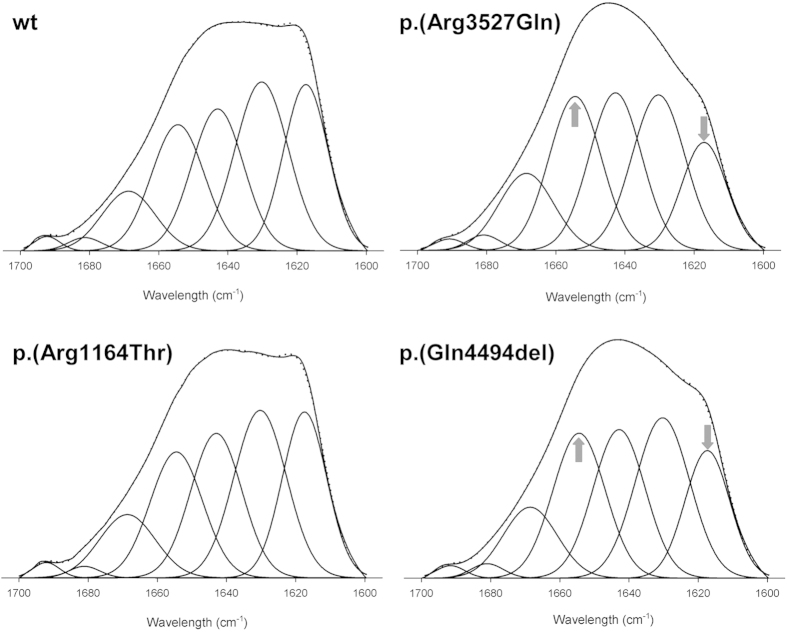
Amide I band decomposition of LDL carrying wt, p.(Arg3527Gln), p.(Arg1164Thr) or p.(Gln4494del) ApoB100 variants. The spectra were obtained in D_2_O buffer at 37 °C as described in Materials and Methods.

In order to analyse by IR spectroscopy the effects produced by p.(Arg3527Gln), p.(Arg1164Thr) and p.(Gln4494del) variants in ApoB100 structure, amide I band of each sample was individually fitted considering the component band position obtained from Fourier derivation and deconvolution, as previously described^25^. These component bands have been assigned to several secondary structures as α-helix, β-turns, sheets or strands, and other unordered structures, usually called random, that typically appear around 1656, 1670, 1630, 1617 and 1642 cm^−1^ in deuterated media, respectively^16^,^24^. In LDL, β -strands are defined as segments embedded inside the particle monolayer, which are characterized by a band at a lower frequency than canonical extended structure (16). The mean secondary structure composition obtained for wt ApoB100 was characterized by a high content in β-structures (>50%) with α-helix content of about 20% (Table 1), in agreement with previous IR studies^24^,^25^. ApoB100 p.(Arg3527Gln) variant showed a reduced content in β-strands (around 3%) with slight increase in α-helixes (2%), similarly to that occurring in p.(Gln4494del) variant in which deletion of Gln at 4494 position produced a clear change in amide I band shape (Fig. 4) mainly resulting in a decrease of β-strands, from 22% to 15%, and in approximately 4% increase in α-helix contribution (Table 1). In contrast, p.(Arg1164Thr) variant showed an amide I band decomposition similar to wt (Table 1), as expected from the similarity of their band shapes (Fig. 4).

## Discussion

FH is one of the most prevalent genetically inherited disorders that leads to greatly increased LDL levels over a lifetime and development of early coronary artery disease^27^. The mechanisms underlying the process of atherosclerotic changes of the vessel wall have been extensively addressed both clinically and experimentally and, searching the APOB gene for new pathogenic variants has disclosed few alterations reported as functionally defective^3^,^8^,^9^,^10^,^11^,^12^,^28^.

In the present work we sought to understand the loss of functionality previously shown for p.(Arg1164Thr) and p.(Gln4494del) ApoB variants^8^. We have used a biophysical approach to determine LDL binding, uptake, particle size and secondary structure of LDL containing p.(Arg3527Gln), p.(Arg1164Thr) and p.(Gln4494del) ApoB variants. A major limitation in the present study is that LDL characterization is based on samples obtained from heterozygous mutation carriers. Nevertheless, the validity of this work is strengthened by previous *in vivo* studies showing that in heterozygous patients, particles carrying functional-defective ApoB100 accumulate in plasma, for example, p.(Arg3527Gln) mutant:wild-type LDL ratio was shown to be ~70:30^29^ and p.(Arg50Trp) mutant:wild-type ratio ~75:25^28^. In this study, the ~50% decreased binding and uptake of LDL together with the ~2/3 reduced proliferation of U937 cells when incubated with LDL from heterozygous patients containing p.(Arg1164Thr) or p.(Gln4494del) variants shows an impaired LDL/LDLR recognition in saturable conditions.

It has been suggested that in patients carrying p.(Arg3527Gln) variant, the impaired removal of LDL from plasma promotes the formation of small dense LDL particles (sdLDL)^10^. In the present work, LDL particles obtained from heterozygous patients carrying p.(Arg3527Gln) or p.(Arg1164Thr) variants show a main population of particles of ~27 nm, significantly smaller than the ones carrying wt ApoB100 (~29 nm) and furthermore, the reduced size is even more pronounced in LDL harbouring p.(Gln4494del) ApoB100, with a mean diameter of ~25 nm. Thus, the smaller size found in LDL carrying p.(Arg1164Thr) and p.(Gln4494del) raises the possibility that delipidation processes may occur *in vivo*. It has been shown that the longer residence of LDL in blood allows an extensive activity of certain enzymes such as hepatic lipase (HL) and, cholesterol ester transfer protein (CETP)^30^,^31^. In fact, *in vitro* studies reveal that lipoprotein lipase (LPL) and HL induce alterations in LDL composition mainly characterized by a substantial reduction in the core triglyceride content^32^. It has also been shown that the sequential effects of lipid transfer and lipolysis promote dramatic changes in the mean size of plasma LDL favouring the formation of sdLDL^33^.

Adding onto that, the mutation itself can distort the structure of the binding domain of ApoB100 as described for p.(Arg3527Gln) mutation^34^. The belt conformation of ApoB100 that surrounds the LDL particle is maintained by interaction of Arg3527 with Trp4396^15^, which stabilizes two clusters of basic amino acids ensuring the binding of ApoB100 to LDLR^35^. It has been proposed that replacement of the Arg3527 promotes a conformational change in ApoB100 causing a rearrangement of a number of charged residues rather than loss of a single receptor-interactive residue^36^. The maintenance of these clusters could also be hampered in the case of p.(Gln4494del) variant because the amino acid deletion moves forward one position into the following amino acid. Therefore, the distortion introduced by p.(Gln4494del) may have a central role in the defective binding and the formation of smaller LDL particles. The analysis of these LDL by IR spectroscopy shows marked differences in the secondary structure of p.(Arg3527Gln) and p.(Gln4494del) compared with wt, their β-strands content being lower while their random and α-helix structure contributions slightly higher. It has been described that the reduced content of β-strands may be related with smaller LDLs^37^ and that changes in the intensity and width of the β-strands band detected by IR spectroscopy might be related to more atherogenic LDL^25^. Although, the differences in the % structure may seem small, considering the big size of ApoB100, these results imply a huge structural rearrangement that likely affects the affinity for LDLR. Therefore, these structural changes may account for the impaired affinity of LDL carrying p.(Arg3527Gln) or p.(Gln4494del).

LDL particle diameter and ApoB100 conformation have implications for the binding affinity of ApoB100 to the receptors^38^ thus the observed differences in one of these parameters, if not both, explain the defective activity of the ApoB100 variants characterized in this work. It is noteworthy that p.(Arg1164Thr) mutation barely modifies ApoB100 structure despite the reduced uptake of this lipoprotein, suggesting that Arg 1164 residue is important for LDL interaction with LDLR.

We conclude that changes found in particle size and/or secondary structure composition of ApoB100 underlie the defective binding and uptake of p.(Arg3527Gln), p.(Arg1164Thr) and p.(Gln4494del) variants. The structural analysis of these LDL carrying ApoB variants helps to understand their defective binding to LDLR, and remarks the importance of residues outside the postulated LDLR binding domain for ApoB100 to adopt a correct structure. *In vitro* experiments mimicking delipidation processes of lipoproteins are still in progress in our laboratory to elucidate how these processes affect both particle size and ApoB100 structure and will help to correlate these factors with LDL binding capacity.

## Materials and Methods

### Study samples

Carriers of variants under study and normolipidaemic subjects were asked to provide a new blood sample to repeat previous ApoB studies and to perform the new structure analysis assays^8^. Serum was frozen at −80 °C until the start of the experiments. Peripheral blood lymphocytes were isolated at room temperature using a density gradient method as described before^39^. The FH patients are included in the Portuguese FH Study, the study protocol was approved by the ethics committee of CEIAB of the Basque Country University and by the National Institute of Health Ethics Committee (Portugal), the methods were carried out in accordance with the approved guidelines. All participants have signed the written informed consent.

### Lipoprotein isolation, characterization and labeling

LDL (1.019–1.063 g/mL) was isolated from human serum by density gradient ultracentrifugation^40^. Protein content of LDL was determined according to Lowry protein assay^41^, triglycerides (TG) and LDL cholesterol (LDL-C) were determined by direct enzymatic procedures using the reagent kit supplied by BioSystem (BioSystem S.A., Barcelona, Spain). LDL was labelled with FITC as previously described^40^.

### Lymphocyte LDL uptake and binding assay

Human lymphocytes were cultured in 24-well culture plates (2 × 10^5^ cells/well) for 72 h in lipoprotein-deficient serum and stimulated with anti-CD3/CD28 beads as previously described^39^ to obtain maximum upregulation of LDLR. LDL uptake was determined in lymphoblast incubated for 4 h with 20 μg/mL FITC–LDL at 37 °C and LDL binding was determined incubating the cells with FITC-LDL at 4 °C. After incubation with FITC-LDL, lymphocytes were washed twice in PBS-1%BSA, fixed on 4% formaldehyde for 10 min and washed again twice with PBS-1%BSA. Fluorescence intensities were measured by flow cytometry in a FACSCalibur Flow cytometer and fluorescence of 10,000 events was acquired for data analysis as described before^39^. The results were expressed as the mean fluorescence of activated gated cells, selected in a forward versus side-scatter window. LDLR genotype (wt) of lymphocyte donors was ascertained by sequencing.

### LDL uptake by HepG2 cells

FITC-labeled LDL uptake by HepG2 cells was also determined. HepG2 cells were grown in monolayer at 37 °C in DMEM with 10% (v/v) foetal bovine serum (FBS, Invitrogen), 100 U/mL penicillin/streptomycin. 5 × 10^4^ cells were seeded in 24-well culture plates for 24 h then FITC–LDL uptake was performed as described above for lymphocytes.

### U937 cells proliferation assay

U937 cells (ATCC CRL-1593.2TM) were grown in RPMI with ampicillin/streptomycin and 10% FBS in a 75-cell culture flask, at 2 × 10^5^ viable cells/mL at 37 °C in a humidified atmosphere of 5% CO_2_. Before proliferation assay, cells were seeded in 96-well culture plates for 24 h in RPMI containing 10% LPDS and the assay was started by adding 2 μg/mL LDL. The cells were further incubated for 48 h at 37 °C allowing cell growth. U937 cell proliferation was determined by CellTiter 96wAQueous Non-Radioactive Cell Proliferation Assay as previously described^8^. The proliferation rate in the presence of LDL carrying wt ApoB100 or the three different mutations has been expressed as fold increment of cellular growth compared with basal growth of cells without added LDL. LDL samples were conditioned for IR measurements as previously described^25^.

### LDL size determination

LDL size was determined from 1 mg/mL protein samples by dynamic light scattering in a Nano-S Zetasizer (Malvern Instruments, UK) as previously described^42^. For the study, all LS measurements were performed at 37 °C in triplicate, with 25 runs of 10 seconds each, using a 173° backscatter detection. The detection limit of the assay for zetasizer instrument used in the present study was 0.3 nm to 10 μm. Viscosity and refractive index of PBS as the dispersant were applied to standard operating protocol prior to size determination. The data were analyzed by zetasizer family software

### LDL size determination by negative stain electron microscopy (NS-EM)

For negative stain electron microscopy (NS-EM) a 10 μL drop of lipoprotein solution (100 μg/mL) was placed on a glow-discharged thin carbon-coated 300-mesh copper grid (Cu-300CN; Pacific Grid-Tech, San Francisco, CA). After ~1 min, the excess solution was removed by blotting with filter paper. The grid was washed three times by briefly touching the surface of the grid with a drop (~30 μL) of deionized water on Parafilm and then blotted dry with filter paper. The touching and blotting steps were performed, each with a clean drop of deionised water. One drop (~30 μL/drop) of 1% (w/v) uranyl acetate (UA) (pH 4.6) solution were applied on Parafilm, and the excess solution was removed by blotting similarly. The grid remained in contact with the last UA drop with the sample side down for 1–3 min in the dark before excess stain was removed and the sample was air dried at room temperature.

Particle size was determined by measuring Feret diameter, individual particle images were selected, picked automatically and manually checked to remove overlapping or damaged particles. More than 1600 particle images from micrographs of each condition were used for statistical analysis of particle size distribution.

### Infrared spectroscopy

LDL samples were conditioned for IR measurements as previosly described^25^. Briefly, isolated LDL particlces were concentrated on Microcon centrifugal filters (Millipore) to a final concentration of 8–10 mg/mL and extensively dialyzed against deuterated PBS buffer at 4 °C in closed container. The buffer was changed several times until completed H_2_O-D_2_O exchange was achieved. Samples were placed in 25 μm carved calcium fluoride windows. Infrared spectra were recorded at 37 °C in a Nicolet Nexus 5700 spectrometer equipped with a MCT detector using a Peltier cell (TempCon, Bio Tools). Buffer subtraction was performed by using the buffers of the last dialysis step as reference and analyzed by Fourier deconvolution and derivation in order to define the number and position of constituent bands of the amide I band. Baseline corrected spectra were fitted using two step procedure as previously described^43^ and considering Gaussian band shape for all component bands^24^.

### Statistical analysis

All measurements were independently performed at least 3 times, with n=3 unless otherwise stated, and results are presented as mean ± s.d. Levels of significance were determined by a two-tailed Student’s t-test, and a confidence level of greater than 95% (p<0.05) was used to establish statistical significance.

## Additional Information

**How to cite this article**: Fernández-Higuero, J. A. *et al*. Structural analysis of APOB variants, p.(Arg3527Gln), p.(Arg1164Thr) and p.(Gln4494del), causing Familial Hypercholesterolaemia provides novel insights into variant pathogenicity. *Sci. Rep*. **5**, 18184; doi: 10.1038/srep18184 (2015).

## Figures and Tables

**Table 1:** Secondary Structure content of LDL carrying wt, p.(Arg3527Gln), p.(Arg1164Thr) or p.(Gln4494del) ApoB100 variants.

Structure	wt ApoB100	p.(Arg3527Gln)	p.(Arg1164Thr)	p.(Gln4494del)
β-Turn	11.0 ± 0.7	12.0 ± 0.8	11.3 ± 0.5	13.4 ± 0.7
α-helix	19.8 ± 1.1	21.6 ± 1.4*	19.2 ± 0.5	24.2 ± 1.4*
Random	21.7 ± 0.4	22.4 ± 0.6	21.6 ± 0.2	23.6 ± 0.5
β Sheet	25.8 ± 0.3	25.5 ± 0.2	25.3 ± 0.4	24.3 ± 2.1
β Strand	21.7 ± 1.8	18.6 ± 1.7*	22.6 ± 1.0	14.5 ± 2.3*

Values shown represent the mean ± standard deviation. *p < 0.01 compared with wt ApoB100.
